# Broncho-Vesiculitis and Bronchial Rheumatism

**Published:** 1874-05

**Authors:** L. B. Anderson

**Affiliations:** Virginia


### BRONCHO-VESICULITIS AND BRONCHIAL RHEUMATISM.

BY L. B. ANDERSON, M.D., OF VIRGINIA.

Having, on two occasions, been the subject of bronchitis in two forms, not mentioned, that I have seen, in any medical work, I hope I may be pardoned the necessary egotism of this paper.

During the winter of 1861, I was actively engaged in an extensive and laborious country practice. There was an unusual amount of sickness in my field, necessitating much exposure both day and night to the very inclement weather. Early in February I had a severe pharyngitis, which was soon followed by laryngitis, and that by bronchitis. During this time I was compelled to stop for a day or two to obtain relief. About the fourth of March, however, all the usual indications of bronchial inflammation save dyspnœa, subsided. There was neither pain or soreness in taking a deep inspiration, but the most oppressive and alarming sense ot suffocation. My pulse was feeble, and when still but little accelerated, the slightest exertion, however, hurried it into a rapid and tumultuous tremor, increasing the sense of suffocation to a most painful degree. There was not the slightest inclination to expectorate, but a feeling of perfect dryness from the nasal orifice to the extremist bronchial ramification. In the mean time the whole surface of the body was bathed in a profuse perspiration, and though I was swathed in flannel and kept my room at a temperature bordering on 100° Farenheit; I was constantly chilly; my liver was torpid; bowels constipated; urine high-colored acrid and of a strong garlic odor. I felt that a full cough and free expectoration would relieve me amazingly.

I was cupped, bled, blistered, pustulated. I took mercury, antimony, ipecac, senega, squill and potass. The treatment was continued actively for several weeks, and then with more or less activity for nearly two years. During which time I was examined by many eminent physicians, who could detect no abnormal sounds in my lungs, and were unable to ascertain a pathological condition which would account for the physical state. It was during the busy whirl of war, and the confinement for nearly two years almost constantly to my room, while the whole world seemed ebbing to and fro in ceaseless agitation, drove me to desperation. Though scarcely able to walk unassisted a hundred yards, I went to Richmond and inlisted as a private in a battery, which was about to leave for active operations in western Virginia. I was sent to a surgeon for examination and he could detect no pulmonary disease. While perfectly passive my breathing and pulse were quite natural, but the slightest exertion would accelerate, both to a strange frequency and cause the perspiration to pour from every pore. The doctor was utterly confounded and declared that I was unfit for service, and had better go home and take iron.

I then disclosed to him my status and gave him what I had decided was the pathological condition giving rise to these unusual symptoms, thanking him for his kind advice, and assuring him that I had no doubt that a few doses of iron would terminate my existence.

Returning home, I organized a scouting party which was kept in pretty active employment by the frequent raids which at that day were made into my section. In the course of a few weeks I found myself laboring under the most inveterate jaundice I ever saw. So terrible was the pruritus that I dared not lie on a bed for two months, and though I used the whole catalogue of jaundice remedies, I found no relief until I resorted to the hot bath, and small doses of gamboge and aloes. At the end of eight weeks I was convalescent, and to my great delight I found my lungs almost as sound as ever they were. I must not omit to state, however, that during this attack of jaundice, I was reduced to the merest skeleton, being literally nothing but skin and bones—my hair became almost white, and my whole constitution seemed to have undergone a radical change.

This is a correct history of my case, with all its essential characteristics. The question now arises, what was the pathological condition, from which it sprang? I answer VESICULAR BRONCHITIS.

The inflammation traveling from above downwards, finally reached the air vesicles at the extreme termini of the bronchial tubes, and before measures were employed for its relief, it had produced so much thickening as to preclude in a great measure the action of the air on the blood. There being no inflammation in or exudation from the lining of the bronchial tubes, there were none of the usual signs of bronchitis. The vesicles in the greater part of the right lung, and in no small part of the left being impervious to air, produced the dyspnœa and perfrigeration, and acceleration of pulse in exercise. The wasteing away of the system and the great activity of the absorbents during the jaundice, reduced the vesicles to their nomal status, and that which nearly terminated my existence, restored me to health.

I have seen several cases of the kind since, and am just now recovering from another attack myself. It is worthy of careful study, and prompt attention, for only a few days will result in such thickening of the vesicles and impairment of the capacity of the lungs, as to foil your best exertions to cure. Active purging, cupping, blistering, and the use of calomel *early* will generally insure relief.

The second form of bronchial disease, of which I have recently been a subjet was marked by the following characteristics: A painful sense of oppression, stricture across the chest, an entire absence of all exhiliration and refreshment from taking a deep inspiration, a sense of unrelief in inspiration as though the air passes through passive tubes, which had no closed extremities, and consequently made no sensible impression upon their delicate lining. In a word, I felt as though *the bottom had fallen out of my lungs.* There was a constant aching very similar to tooth-ache all through the right lung, and a constant disposition to take a deep inspiration. There was no cough, no expectoration, no acute pain, no fever. The urine was high colored and loaded with sediment.

I was freely cupped over the chest; took active purges; used syrup of sanguinaria freely, and kept warmly wrapped in bed, as I felt chilly all the while.

On the third day I awoke early in the morning with entire relief from all the pectoral troubles, but I was suffering with quite a severe pain in my face and head. This in turn passed off during the following night. The following day I rode out in a bleak western wind, and that eveniug all the pulmonary symptoms returned with increased violence. I had some soreness in the bronchia and the aching throughout the right lung was distressing. I was sleepless, feverish, and restless all night. The following day I took sanguinaria freely, cupped heavily, and took a purge. Not having slept for twenty-four hours, I fell asleep at ten o'clock at night and slept profoundly till five o'clock in the morning, awaking entirely relieved. But found myself laboring under a severe lumbago. What was the matter? I answer "bronchial rheumatism." Such cases I had sometimes seen in others, but confess I never had a clear and distinct idea of its pathology before.

